# Supplementary material for: Diet of Andean Leaf‐Eared Mice (Phyllotis) Living at Extreme Elevations on Atacama Volcanoes: Insights From Metagenomics, DNA Metabarcoding, and Stable Isotopes
Source: Ecol Evol. 2024 Nov 18;14(11):e70591. doi: 10.1002/ece3.70591 (PMC11573726; doi:10.1002/ece3.70591)
Supplement: Supplementary file 1 — Table S1. [file ECE3-14-e70591-s001.pdf]

Supplemental Information for:

Diet of Andean leaf-eared mice (*Phyllotis*) living at extreme elevations on Atacama volcanoes: insights from metagenomics, DNA metabarcoding, and stable isotopes

Claudio Quezada-Romegialli, Marcial Quiroga-Carmona, Guillermo D’Elía, Chris Harrod, Jay F. Storz

Tables S1-S6

Table S1. Input, filtered, denoised forward, denoised reverse, merged and non-chimeric reads for trnL P6 loop primers per sample.

Sample	Input	Filtered	DenoisedF	DenoisedR	Merged	Nonchim
Extraction blank	17	10	9	7	7	7
stomach	65.723	63.552	63.537	63.504	61.611	61.491
cecum	85.849	71.919	71.819	71.622	69.401	68.688
C1	87.333	82.656	82.563	82.579	80.032	80.032
C2	66.839	64.199	64.185	64.186	62.649	62.649
C3	70.175	67.173	66.928	67.042	64.984	64.984
C4	69.287	66.445	66.367	66.427	64.282	64.282
C5	64.639	62.033	61.992	61.949	59.975	59.836
C6	72.401	69.428	69.406	69.400	67.528	67.528
C7	77.599	73.226	73.059	73.050	70.801	70.797
C8	71.317	64.527	64.444	64.344	62.261	62.261
C9	75.808	72.965	72.928	72.883	71.345	70.517
C10	66.679	59.690	59.569	59.357	57.510	57.475
C11	59.982	56.208	56.078	56.082	54.377	54.377
C12	66.209	62.673	62.581	62.557	60.312	60.312
**TOTAL**	**999.857**	**936.704**	**935.465**	**934.989**	**907.075**	**905.236**

Table S2. Input, filtered, denoised forward, denoised reverse, merged and non-chimeric reads for ITS2 primers per sample.

Sample	Input	Filtered	DenoisedF	DenoisedR	Merged	Nonchim
stomach	186.662	51.882	51.729	51.665	43.165	40.150
cecum	192.646	38.775	38.675	38.576	28.196	27.172
C1	52.978	12.036	11.994	11.989	8.971	8.969
C2	131.565	23.473	23.366	23.276	18.090	18.049
C3	96.694	21.695	21.650	21.587	16.442	16.436
C4	123.788	24.462	24.379	24.344	18.907	18.713
C5	69.594	15.980	15.914	15.912	11.709	11.289
C6	104.771	23.918	23.846	23.812	18.447	18.441
C7	126.643	32.258	32.161	32.020	24.754	24.652
C8	122.089	32.204	32.076	31.980	25.172	24.829
C9	95.100	31.228	31.127	31.081	25.353	25.026
C10	92.587	22.065	21.927	21.975	16.832	16.639
C11	85.107	22.338	22.236	22.172	17.711	17.570
C12	69.403	18.381	18.328	18.274	14.278	14.036
**TOTAL**	**1.549.627**	**370.695**	**369.408**	**368.663**	**288.027**	**281.971**

Table S3. Input, filtered, denoised forward, denoised reverse, merged and non-chimeric reads for p23SrV primers per sample.

Sample	input	filtered	denoisedF	denoisedR	merged	nonchim
Extraction blank	116	18	15	15	0	0
stomach	164.960	34.357	34.303	34.285	18.684	18.608
cecum	159.183	29.170	28.900	28.876	24.067	23.714
C1	105.590	18.439	18.325	18.292	14.736	14.717
C2	108.051	10.336	10.249	10.232	8.665	8.662
C3	144.892	34.201	34.058	33.990	26.655	26.598
C4	91.943	15.960	15.854	15.791	12.904	12.859
C5	126.028	18.813	18.679	18.668	15.074	14.965
C6	153.258	28.918	28.684	28.567	23.668	23.513
C7	132.896	19.196	18.973	18.880	15.059	14.903
C8	158.723	30.256	29.972	29.933	24.503	24.099
C9	168.609	31.725	31.394	31.382	25.961	24.907
C10	140.728	19.920	19.727	19.650	16.087	15.898
C11	119.463	24.898	24.625	24.547	19.867	19.658
C12	160.913	38.832	38.473	38.418	33.246	32.837
**TOTAL**	**1.935.353**	**355.039**	**352.231**	**351.526**	**279.176**	**275.938**

Table S4. Input, filtered, denoised forward, denoised reverse, merged and non-chimeric reads for ITS1 primers per sample.

Sample	input	filtered	denoisedF	denoisedR	merged	nonchim
Extraction blank	30	14	5	3	3	3
stomach	85.919	60.669	60.285	60.359	58.840	57.953
cecum	85.667	55.148	54.810	54.640	53.480	53.017
C1	100.641	57.430	57.176	57.123	54.667	50.079
C2	149.940	97.006	96.686	96.554	90.605	85.856
C3	95.950	61.266	61.019	60.872	58.095	57.108
C4	88.134	57.532	57.335	57.250	54.658	53.568
C5	64.992	37.085	36.865	36.754	35.340	35.011
C6	69.510	46.261	45.984	45.761	41.148	38.846
C7	93.476	52.639	52.279	52.107	49.869	45.908
C8	119.736	88.616	88.288	88.184	86.796	84.821
C9	120.747	78.783	78.313	78.173	75.869	74.128
C10	98.785	63.102	62.659	62.609	60.707	59.641
C11	141.305	74.558	74.107	73.899	64.854	62.434
C12	183.546	100.844	100.256	100.094	97.551	95.722
**TOTAL**	**1.498.378**	**930.953**	**926.067**	**924.382**	**882.482**	**854.095**

Table S5. Input, filtered, denoised forward, denoised reverse, merged and non-chimeric reads for COI (metazoan) primers per sample.

Sample	input	filtered	denoisedF	denoisedR	merged	nonchim
Extraction blank	1.816	1.117	1.116	1.114	0	0
stomach	166.575	79.737	79.626	79.668	1.413	1.412
cecum	133.126	71.365	71.264	71.288	840	819
C1	101.655	55.856	55.789	55.840	874	873
C2	138.917	78.527	78.461	78.501	669	669
C3	130.863	79.014	78.922	78.954	2.610	2.610
C4	90.177	32.118	32.076	32.085	796	796
C5	169.215	101.419	101.294	101.365	2.129	2.129
C6	155.282	86.844	86.756	86.758	1.685	1.685
C7	146.412	83.350	83.184	83.233	3.704	3.604
C8	169.047	92.995	92.885	92.918	1.821	1.811
C9	150.635	73.805	73.715	73.746	725	725
C10	161.401	90.767	90.645	90.656	3.411	3.411
C11	155.326	88.127	87.905	87.800	3.250	3.230
C12	135.045	69.068	69.003	69.010	1.802	1.802
**TOTAL**	**2.005.492**	**1.084.109**	**1.082.641**	**1.082.936**	**25.729**	**25.576**

Table S6. Input, filtered, denoised forward, denoised reverse, merged and non-chimeric reads for COI (invertebrates) primers per sample.

Sample	input	filtered	denoisedF	denoisedR	merged	nonchim
Extraction blank	30	14	5	3	3	3
stomach	85.919	60.669	60.285	60.359	58.840	57.953
cecum	85.667	55.148	54.810	54.640	53.480	53.017
C1	100.641	57.430	57.176	57.123	54.667	50.079
C2	149.940	97.006	96.686	96.554	90.605	85.856
C3	95.950	61.266	61.019	60.872	58.095	57.108
C4	88.134	57.532	57.335	57.250	54.658	53.568
C5	64.992	37.085	36.865	36.754	35.340	35.011
C6	69.510	46.261	45.984	45.761	41.148	38.846
C7	93.476	52.639	52.279	52.107	49.869	45.908
C8	119.736	88.616	88.288	88.184	86.796	84.821
C9	120.747	78.783	78.313	78.173	75.869	74.128
C10	98.785	63.102	62.659	62.609	60.707	59.641
C11	141.305	74.558	74.107	73.899	64.854	62.434
C12	183.546	100.844	100.256	100.094	97.551	95.722
**TOTAL**	**1.498.378**	**930.953**	**926.067**	**924.382**	**882.482**	**854.095**
